# Mortality in IgA Nephropathy: A Long-Term Follow-Up of an Eastern European Cohort

**DOI:** 10.3390/medicina60020247

**Published:** 2024-01-31

**Authors:** Gabriel Ștefan, Adrian Zugravu, Simona Stancu

**Affiliations:** 1 Nephrology Department, University of Medicine and Pharmacy “Carol Davila”, 050474 Bucharest, Romania; adzugravu@yahoo.com (A.Z.); simonastancu2003@yahoo.com (S.S.); 2 Nephrology Department, “Dr. Carol Davila” Teaching Hospital of Nephrology, 010731 Bucharest, Romania

**Keywords:** immunoglobulin A nephropathy, prognosis, mortality, end-stage kidney disease, risk factor

## Abstract

*Background and Objectives*: IgA nephropathy (IgAN), the most common primary glomerulonephritis, has been extensively studied for renal outcomes, with limited data on patient survival, particularly in Eastern Europe. We aimed to investigate the long-term survival rate of patients with IgAN and the associated risk factors in an Eastern European cohort. *Materials and Methods*: We conducted a retrospective analysis of 215 IgAN patients (median age 44, 71% male) diagnosed at a Romanian tertiary center between 2010 and 2017. We assessed clinical and pathological attributes, including the Charlson comorbidity index, the prevalence of diabetes, renal function, and treatment with renin-angiotensin-system inhibitors (RASIs). *Results*: Over a median 7.3-year follow-up, 20% of patients died, mostly due to cardiovascular diseases. Survival rates at 1, 5, and 10 years were 93%, 84%, and 77%, respectively. Deceased patients had higher Charlson comorbidity index scores, greater prevalence of diabetes, and poorer renal function. They were less frequently treated with RASIs and more frequently reached end-stage kidney disease (ESKD). *Conclusions*: We report a 20% mortality rate in our Eastern European IgAN cohort, primarily due to cardiovascular diseases. Death correlates with increased age, comorbidity burden, decreased renal function at diagnosis, and the absence of RASI use. RASI treatment may potentially improve survival, highlighting its importance in managing IgAN.

## 1. Introduction

IgA nephropathy (IgAN), the most frequently diagnosed primary glomerulonephritis via kidney biopsy globally, affects up to 350,000 individuals each year and has a point prevalence of 2.53 per 10,000 patients of all ages in Europe [1,2,3]. Given the limited availability of kidney biopsies, IgAN often remains undetected for extended periods, leading to an increased risk of end-stage kidney disease (ESKD). The disease’s progression is typically slow, but the variability in clinical and pathological features makes the prognosis unpredictable [4]. While kidney survival rates range from 60% to 90% over ten years, up to 40% of patients may reach ESKD within 20 years of diagnosis [5,6]. Despite extensive research into ESKD risk factors, our understanding of long-term mortality risks associated with IgAN remains insufficient [7,8,9]. Thus, few studies have specifically tracked the mortality risk associated with IgAN, reflecting a knowledge gap in this critical area. Geographical disparities in IgAN incidence and prevalence are evident, with notably higher values reported in Asia [1]. As a result, mortality rates, causes, and risk factors are also expected to vary across different geographical regions. Mortality data related to IgAN have been published only from a limited range of countries, including Sweden, Norway, Korea, the United States, the United Kingdom, and Taiwan [10,11,12,13,14,15]. IgAN was linked to reduced life expectancy, with most excess mortality risk emerging post-kidney failure [10]. The median time to ESKD varied across countries (23–32 years), with post-kidney replacement therapy median survival being 8 years in the VALIGA cohort and 10 years in Sweden [16]. Notably, Norway and Sweden, with their universal, publicly financed healthcare systems, reported the longest overall survival, suggesting that healthcare differences between countries may influence outcomes [17]. However, a significant lack of information exists for Eastern Europe, where no such reports have been released.

Therefore, we aimed to comprehensively evaluate the survival and its predictive factors in patients with IgA nephropathy, alongside analyzing renal outcome and indicators, at a large tertiary center in Romania.

## 2. Materials and Methods

### 2.1. Study Population

We conducted a retrospective, observational study on all patients with biopsy-proven primary IgAN from 2010 to 2017 at a tertiary department of nephrology in Romania, Eastern Europe. Those whose kidney biopsy specimens were inadequate for MEST-C classification (n = 4), with secondary cause of IgAN (n = 42) [18,19], with insufficient clinical data (n = 3), or with less than 12 months follow-up (n = 9) were excluded from the analysis, leaving a final cohort of 215 patients.

This study was in accordance with the Declaration of Helsinki and this study was approved by the local Ethics Committee.

### 2.2. Data Collection

The clinical variables obtained by reviewing the patient’s electronic medical records at the time of kidney biopsy were age, gender, Charlson comorbidity index [20], obesity (defined as a body mass index > 30 kg/m^2^), diabetes mellitus and arterial hypertension (defined as blood pressure > 140/90 mmHg or use of antihypertensive agents), therapy with renin-angiotensin-system inhibitors (RASIs), and immunosuppressive medication.

Laboratory data included serum creatinine, estimated glomerular filtration rate (eGFR, calculated by CKD-EPI equation), serum albumin, serum cholesterol, and triglycerides, proteinuria (g/g creatinine), and hematuria (cells/mm^3^).

The indication for immunosuppressive therapy was at the discretion of the nephrologist in charge.

### 2.3. Pathological Measurements

The diagnosis of IgAN was based on light microscopy, immunofluorescence (dominant IgA in the mesangium), and electron microscopy (para-mesangial electron-dense deposits). All the kidney biopsy specimens were reviewed by an experienced nephropathologist blinded to the clinical and laboratory data and were scored according to MEST-C Oxford [21].

### 2.4. Study Endpoints

The primary endpoint of our study was all-cause mortality, while the secondary endpoint was the onset of ESKD, defined as the initiation of kidney replacement therapy (either hemodialysis or peritoneal dialysis) or undergoing renal transplantation. The follow-up period for patients extended until either they reached one of these endpoints, or until 1 January 2022, whichever occurred first.

### 2.5. Statistical Analysis

Continuous variables were presented as mean or median with interquartile range (IQR) after testing normality with the Shapiro–Wilk test, while categorical variables were presented as percentages.

Differences between groups were analyzed using the student t-test or Kruskal–Wallis test, depending on the distribution of the variables, and the Pearson χ^2^ test was used for categorical variables.

The probability of event-free survival was assessed by Kaplan–Meier method.

Univariate and multivariate (Cox proportional hazard ratio) analyses were performed to identify independent predictors of the primary and secondary endpoints. The results of Cox regression analyses are expressed as a hazard ratio (HR) and 95% confidence interval (95% CI).

In all analyses, *p* values are two-tailed and all *p* values less than 0.05 were considered statistically significant.

SPSS version 20 (Chicago, IL, USA) and GraphPad Prism version 9.5.1 (GraphPad Software, La Jolla, CA, USA) were used for statistical analyses.

## 3. Results

In this study, we followed a cohort of 215 patients diagnosed with IgAN for a median duration of 88 months (95% CI: 84.8, 91.8). Out of the total patients, 43 (20%) died during the follow-up period. The median age of the total cohort was 44 years, with 71% being male. The median Charlson comorbidity index was 2, and the prevalence of obesity, diabetes mellitus, and hypertension was 34%, 9%, and 80%, respectively. Kidney function parameters revealed a median eGFR of 40.9 mL/min/1.73 m^2^ and a median proteinuria of 1.24 g/g creatinine (Table 1).

When compared to the survivors, deceased patients were significantly older and had an increased prevalence of diabetes mellitus and a higher Charlson comorbidity index. Deceased patients also had significantly higher serum creatinine levels, lower eGFR, and more hematuria. There was no significant difference in proteinuria between the two groups (Table 1).

In examining the histological findings, we noted that the deceased patients presented with a greater degree of tubular atrophy and interstitial fibrosis (T1/2), and their MEST-C scores were significantly higher. Conversely, the presence of mesangial hypercellularity (M1), endocapillary hypercellularity (E1), segmental glomerulosclerosis (S1), and crescents (C1/2) did not display any significant differences between the two patient groups (Table 1).

Regarding the treatment, fewer patients who died (37%) were treated with renin-angiotensin-system inhibitors compared to those who lived (68%, *p* < 0.001). Furthermore, the initiation of kidney replacement therapy was notably more frequent in the deceased patient group (47% versus 30%, *p* = 0.04). The administration of immunosuppression therapy, however, did not display a statistically significant difference between the two groups (Table 1).

The main causes of death in the studied IgAN cohort were cardiovascular diseases (60%), followed by infectious (19%), gastroenterological (12%), and neoplastic (9%) diseases.

**Figure 1:**
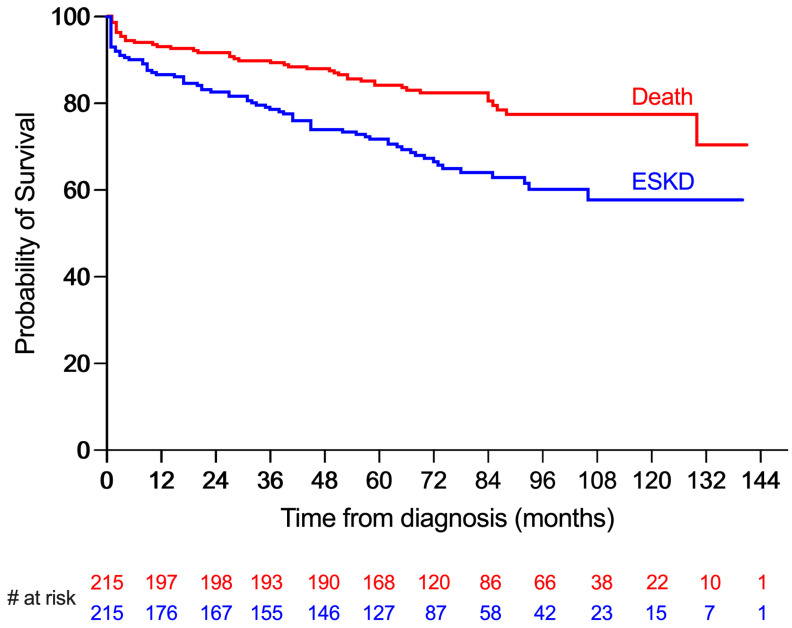
Cumulative survivals: survival in months from diagnostic kidney biopsy to date of death (red line) and to end-stage kidney disease (ESKD), defined as renal replacement therapy initiation or renal transplantation (blue line). The numbers of at-risk patients at each 12-month interval are shown below for both survival curves.

The mean survival of the entire cohort was 117.5 months (95%CI: 111.2, 123.8), and the mean kidney survival was 98.4 months (95%CI: 90.5, 106.2). Survival rates at 1 year, 5 years, and 10 years were 93%, 84%, and 77%, respectively, and kidney survival rates at 1 year, 5 years, and 10 years were 87%, 72%, and 58%, respectively (Figure 1).

In the univariate Cox regression analysis, all-cause mortality was significantly associated with several factors: advancing age, an increased Charlson comorbidity index, the presence of diabetes mellitus, lower eGFR, increased proteinuria, the presence of hematuria, higher cholesterol, lower serum albumin, higher MEST-C score, and the absence of RASI. However, in the multivariate analysis, only an increased Charlson comorbidity index, lower eGFR, and the absence of RASI retained their statistical significance (Table 2).

As for ESKD, the univariate analysis identified age, male sex, a higher Charlson comorbidity index, the presence of diabetes mellitus, hypertension, lower eGFR, increased proteinuria, higher triglycerides, lower serum albumin, higher uric acid, an increased MEST-C score, and the absence of RASI as significantly associated factors. The multivariate analysis, however, showed that male sex, the presence of diabetes mellitus, lower eGFR, increased proteinuria, and the absence of RASI use were significantly predictive of ESKD (Table 2).

## 4. Discussion

In our study involving 215 patients with IgAN followed for a mean of 7.3 years, we observed a 20% mortality rate, with cardiovascular diseases being the primary cause of death. Deceased patients were generally older, with more comorbidities and worse kidney function. The key predictors of all-cause mortality included advanced age, higher Charlson comorbidity index, and lower eGFR. Notably, the use of renin-angiotensin-system inhibitors emerged as a potential protective factor, being associated with better survival and a lower risk of ESKD.

In the most comprehensive study on IgAN mortality to date, Jarrick et al. examined 3622 biopsy-proven IgAN patients in Sweden, including 70% men and an average diagnosis age of 34.9. Throughout the median follow-up period of 13.6 years, 577 patients (16%) died. The study reported a mortality hazard ratio of 1.53 compared to matched controls, with the highest mortality and ESKD risks occurring in the first year post-diagnosis, leading to an estimated life expectancy reduction of 6 years. Notably, mortality rates were not elevated before ESKD development compared to family or population controls but were higher in patients receiving steroids or immunosuppressants. Conversely, renin-angiotensin-system inhibitors or statin users did not exhibit an increased mortality risk. The study also reported a significant 100-fold increased ESKD risk in IgAN patients versus controls, and a threefold elevated ESKD risk in the first-degree relatives of IgAN patients [10]. Compared to this study, our findings also indicated a similar mortality rate due to cardiovascular disease. However, our study involved a significantly older population, with an average age of 44 years, as opposed to an average of 34.9 years (Table 3).

Our study reported a 20% mortality rate, identical to that found in a similar population in the UK, as evidenced by comparable eGFR, proteinuria, and MEST-C class distribution (Table 3) [14]. However, our study had a significantly longer follow-up time of 7.3 years compared to the UK’s 4.3 years [14]. This mortality rate aligns with other European reports, though it is lower than that reported in the US and higher than in Asian studies from Korea and Taiwan (Table 3). A key factor likely contributing to these mortality rate disparities could be the renal function at the time of diagnosis, which is typically lower in European studies compared to those from Asia (Table 3). Therefore, the discrepancies in mortality rates may reflect differences in diagnostic practices or biopsy policies among different regions. This suggests later referrals to nephrologists in Europe and underscores the value of systematic mass urine screenings implemented in certain Asian regions (such as Hong Kong, Japan, Korea, and Singapore) [1,22].

Similarly to previous reports from Sweden and Norway [10,11], ESKD occurred three times more frequently than pre-ESKD deaths in our population. Moreover, mortality was almost double after kidney replacement therapy initiation (28% versus 16%, *p* = 0.03). Our study supports the findings by Jarrick et al. [10] and Knoop et al. [11], demonstrating that the increased mortality risk in IgAN predominantly occurs after ESKD onset. This contradicts large-scale studies on generalized chronic kidney disease (CKD), where mortality risk elevates even before reaching ESKD and is particularly pronounced in younger patients [23,24,25,26]. The reason for this discrepancy between IgAN and general CKD is unclear, but it appears that younger IgAN patients with no extrarenal organ involvement or serious comorbidities at diagnosis can withstand the progression of CKD, succumbing only during ESKD [27]. This challenges the universal applicability of mortality risk observed in generalized CKD studies, which often group diverse kidney diseases under the ‘generic’ CKD label.

Numerous studies have shown that both a reduced eGFR and increased proteinuria escalate the risk of cardiovascular disease [23,24,25]. As IgAN is incurable, all patients ultimately progress to CKD, which several studies identify as a risk equivalent to coronary heart disease. In line with this, our study found that a lower baseline eGFR was associated with a heightened mortality rate, primarily due to cardiovascular disease. Like in two previous reports [10,13], the absence of RASI was also linked to an increased mortality in our IgAN population (Table 3). RASIs have been proven to decrease morbidity and mortality in patients with heart failure and reduced ejection fraction (HFrEF) [28,29]. Thus, robust evidence supports the use of RASI in patients diagnosed with stages CKD 1–3 and HFrEF [30], which is the most frequently observed clinical scenario at the time of an IgAN diagnosis.

Although a link between the MEST-C score and mortality was evident in the univariate analysis, this relationship was not sustained in the multivariate analysis. Of the two prior studies that assessed the connection between MEST-C classification and death, only one study from Taiwan found an association between the presence of crescents and mortality (Table 3) [15,31].

Few studies specifically focus on mortality as a separate endpoint in IgAN (Table 3), often combining it with ESKD in a composite endpoint and not detailing each event individually [32,33]. Addressing vital survival separately is crucial, particularly given IgAN’s impact on a younger demographic and its significant social and economic effects [34]. Additionally, survival may vary based on a country’s healthcare policies, with active screening in Asian populations showing improved outcomes [34]. Understanding regional variations in IgAN patient survival is essential, especially with the development of various treatment classes in advanced trials. These include endothelin receptor antagonists, B-cell-directed treatments, complement system inhibitors, SGLT2 inhibitors like dapagliflozin, and immunomodulatory drugs such as hydroxychloroquine [35,36,37].

The strengths of our study include its real-life setting, enhancing its relevance and the applicability of the findings to everyday clinical practice. Moreover, our study is comprehensive, considering the entire spectrum of risk factors for IgAN progression, including histological factors. We utilized hard endpoints, which contributes to the reliability of our results. Furthermore, the lengthy follow-up period of our study allows for a more thorough understanding of disease progression and long-term outcomes. However, there are also limitations to consider. Our study was unicentric, involving a relatively limited population, which might restrict the generalizability of our results to broader or more diverse populations.

## 5. Conclusions

In conclusion, our study, the first of its kind from Eastern Europe, reports a 20% mortality rate in IgAN patients, chiefly attributed to cardiovascular disease. Risk factors include age, high comorbidity burden, and reduced eGFR at diagnosis, while the use of renin-angiotensin-system inhibitors potentially improves survival. However, the findings, though significant, are based on a single-center and a limited population, necessitating further validation through larger, multicenter studies.

## Figures and Tables

**Table 1:** Clinical, histological, and treatment findings at the time of diagnostic kidney biopsy and the relationship with the mortality.

	All N = 215	Alive n = 172	Dead n = 43	*p*
Age (years)	44 (34–58)	42 (34–52)	60 (55–64)	<0.001
Male sex (%)	71	67	84	0.03
Charlson comorbidity index	2 (0–4)	2 (0–2)	5 (3–7)	<0.001
Obesity (%)	34	33	35	0.8
Diabetes mellitus (%)	9	4	26	<0.001
Hypertension (%)	80	78	86	0.2
Serum creatinine (mg/dL)	1.79 (1.30–2.74)	1.64 (1.21–2.36)	3.39 (2.15–5.32)	<0.001
eGFR (mL/min/1.73 m^2^)	40.9 (22.7–61.1)	46.1 (30.3–63.6)	18.0 (9.0–32.5)	<0.001
Proteinuria g/g creatinine	1.24 (0.62–2.36)	1.25 (0.58–2.23)	1.14 (0.69–3.11)	0.3
Hematuria (cells/mm^3^)	190 (40–250)	183 (35–240)	230 (60–500)	0.01
Cholesterol (mg/dL)	203 (172–250)	207 (174–256)	186 (149–238)	0.04
Triglycerides (mg/dL)	152 (106–242)	148 (106–242)	161 (101–244)	0.7
Serum albumin (g/dL)	4.20 (3.81–4.56)	4.24 (3.90–4.59)	3.85 (3.42–4.30)	<0.001
Uric acid (mg/dL)	6.70 (5.68–8.30)	6.60 (5.47–8.20)	7.00 (6.10–8.68)	0.1
Kidney biopsy				
M1 (%)	60	59	63	0.8
E1 (%)	28	27	35	0.2
S1 (%)	49	46	61	0.08
T1/2 (%)	20/9	17/6	33/21	<0.001
C1/2 (%)	17/6	17/5	19/9	0.5
MEST-C score	2 (1–3)	2 (1–3)	3 (2–4)	<0.01
Treatment (%)				
Immunosuppression therapy	43	42	47	0.5
RASI	62	68	37	<0.001
ESKD (%)	34	30	47	0.04

eGFR, estimated glomerular filtration rate; M1, mesangial hypercellularity; HPF, high power field; E1, endocapillary hypercellularity; S1, segmental glomerulosclerosis; T1/2, tubular atrophy and interstitial fibrosis; C1/2, crescents; ESKD, end-stage kidney disease; RASI, renin-angiotensin-system inhibitors.

**Table 2:** Relationship between baseline variables and all-cause mortality and end-stage kidney disease utilizing univariate and multivariate Cox regression analyses.

	All-Cause Mortality				ESKD			
	Univariate		Multivariate *		Univariate		Multivariate *	
	HR (95%CI)	*p*	HR (95%CI)	*p*	HR (95%CI)	*p*	HR (95%CI)	*p*
Age (years)	1.08 (1.05–1.11)	<0.001	1.02 (0.99–1.06)	0.09	1.02 (1.00–1.04)	0.01	0.98 (0.96–1.01)	0.09
Male sex	2.17 (0.96–4.89)	0.06	-	-	1.88 (1.05–3.37)	0.03	2.36 (1.24–4.49)	<0.01
Charlson comorbidity index	1.60 (1.42–1.80)	<0.001	1.31 (1.09–1.57)	<0.01	1.29 (1.17–1.41)	<0.001	-	-
Obesity	1.09 (0.58–2.06)	0.7	-	-	1.01 (0.62–1.65)	0.9	-	-
Diabetes mellitus	5.13 (2.57–10.25)	<0.001	-	-	3.39 (1.77–6.50)	<0.001	2.28 (1.04–4.97)	0.03
Hypertension	1.70 (0.72–4.05)	0.2	-	-	4.18 (1.68–10.38)	<0.01	-	-
Serum creatinine (mg/dL)	1.36 (1.24–1.49)	<0.001	-	-	1.65 (1.49–1.83)	<0.001	-	-
eGFR (mL/min/1.73 m^2^)	0.95 (0.93–0.97)	<0.001	0.98 (0.96–1.00)	0.04	0.94 (0.92–0.95)	<0.001	0.93 (0.92–0.95)	<0.001
Proteinuria g/g creatinine	1.12 (1.00–1.25)	0.03	-	-	1.28 (1.17–1.39)	<0.001	1.17 (1.07–1.27)	<0.001
Hematuria (cells/mm^3^)	1.26 (1.03–1.53)	0.02	-	-	1.05 (0.90–1.22)	0.4	-	-
Cholesterol (mg/dL)	0.99 (0.98–0.99)	<0.01	-	-	1.00 (0.99–1.00)	0.4	-	-
Triglycerides (mg/dL)	1.18 (0.70–1.99)	0.5	-	-	1.72 (1.15–2.59)	<0.01	1.44 (0.93–2.24)	0.09
Serum albumin (g/dL)	0.35 (0.22–0.56)	<0.001	-	-	0.42 (0.28–0.63)	<0.001	-	-
Uric acid (mg/dL)	1.17 (0.98–1.38)	0.06	-	-	1.38 (1.21–1.57)	<0.001	-	-
MEST-C score	1.38 (1.13–1.69)	0.001	-	-	1.56 (1.32–1.84)	<0.001	-	-
Immunosuppression therapy	0.80 (0.44–1.46)	0.4	-	-	0.68 (0.43–1.09)	0.1	-	-
RASI	0.32 (0.17–0.60)	<0.001	2.21 (1.15–4.28)	0.01	0.51 (0.31–0.81)	<0.01	0.63 (0.37–1.06)	0.08
ESKD	1.64 (0.90–2.98)	0.1	-	-	-	-	-	-

* Multivariate model analysis was performed only for those lesions that were significant in univariate analysis using a backward elimination (Wald). eGFR, estimated glomerular filtration rate; M1, mesangial hypercellularity; E1, endocapillary hypercellularity; S1, segmental glomerulosclerosis; T1/2, tubular atrophy and interstitial fibrosis; C1/2, crescents; ESKD, end-stage kidney disease; RASI, renin-angiotensin-system inhibitors; HR, hazard ratio.

**Table 3:** Comparative data from studies on mortality in IgA nephropathy.

Study, Year	Jarrick et al. [10] 2019	Lee et al. [12] 2012	Knoop et al. [11] 2013	Hastings et al. [13] 2017	Chen et al. [15] 2020	Storrar et al. [14] 2022	Current Study 2023
Country	Sweden	Korea	Norway	USA	Taiwan	UK	Romania
Period of enrolment	1974–2011	1979–2008	1988–2004	1976–2005	2003–2013	2000–2019	2010–2017
N	3622	1364	633	251	388	401	215
Age (years)	34.9	33	39	36.9	40.9	45	44
Men (%)	70.1	50	74	68.5	55	69.6	71
Mean follow-up time (years)	13.6	10.5	11.8	19.3	7.2	4.2	7.3
eGFR (mL/min)	NA	67.6	NA; 32% had <60 mL/min	NA; 53.4% had <60 mL/min	57.1	46.7	40.9
Proteinuria	NA	1.3 g/24 h	NA; 53% had >1 g/24 h	NA; 67% >1 g/24 h	2.4 g/g	1.83 g/g	1.24 g/g
Hypertension (%)	NA	38.7	45	52	NA	57.6	80
Diabetes mellitus (%)	4.3	2	1.6	6	NA	7.5	9
RASI (%)	74	30.6	NA	56	NA	79.6	62
Immunosuppression (%)	48	12.7	NA	19	NA	20.4	43
Mortality (n, %)	577; 15.9%	71; 5.2%	80; 12.6%	97; 39%	12; 3%	79; 19.7%	43; 20%
Main cause of death	Cardiovascular	Neoplasia	Cardiovascular	NA	Infectious	NA	Cardiovascular
ESKD (n, %)	803; 22.2%	277; 20%	146; 23%	132; 53%	77; 19.8%	119; 29.7%	72; 34%
Risk factors associated with mortality in multivariate analysis	Use of IS and other anti-HTA than RASI	Elderly, SBP > 140 mmHg, hypoalbuminemia, malignancy	NA	Advanced age, CKD stage 4,5, P-uria > 3 g/24 h, absence of RASI, CS therapy	Elderly, presence of crescents, low HDL	Elderly, diabetes mellitus, lower eGFR	Elderly, Charlson index, lower eGFR, absence of RASI

CKD, chronic kidney disease; CS, corticosteroids; eGFR, estimated glomerular filtration rate; ESKD, end-stage kidney disease; HTA, arterial hypertension; IS, immunosuppression; NA, not assessed; M1, mesangial hypercellularity; E1, endocapillary hypercellularity; S1, segmental glomerulosclerosis; T1/2, tubular atrophy and interstitial fibrosis; C1/2, crescents; P-uria, proteinuria; RASI, renin-angiotensin-system inhibitors; SBP, systolic blood pressure.

## Data Availability

The data underlying this article will be shared on reasonable request by the corresponding author.
